# KNOWLEDGE OF GENDER-SPECIFIC TYPES OF CANCER AMONG OLDER LATINOS/AS

**DOI:** 10.1093/geroni/igad104.0652

**Published:** 2023-12-21

**Authors:** Iraida Carrion, Malinee Neelamegam, Tania Estapé, Jorge Estapé

**Affiliations:** University of South Florida, Tampa, Florida, United States; University of North Texas Health Science Center, Fort Worth, Texas, United States; Fundación contra el cáncer, Barcelona, Catalonia, Spain; Fundación contra el cáncer, Barcelona, Catalonia, Spain

## Abstract

Given the growing population of Latino/a immigrants 60 years and older and the current lack of relevant data, our study aimed to understand the knowledge of gender-specific types of cancer among older Latinos/as to ensure effective prevention, intervention, and cancer care. Using convenience sampling, 200 individuals identifying as Latino/a were surveyed in Tampa, Florida. Results indicate significant differences in men’s and women’s knowledge of breast cancer and prostate cancer preventative measures. The differences in cancer knowledge among older Latino/a men and women regarding the opposite gender are relevant when providing cancer education to older adults. Providing culturally and linguistically appropriate cancer care and education can help address decision-making, achieve equity, and improve cancer outcomes for older Latinos/as.

